# Semen pH and its correlation with motility and count - A study in subfertile men

**DOI:** 10.5935/1518-0557.20200080

**Published:** 2021

**Authors:** Sanketh Satya Dhumal, Prashanth Naik, Swaminathan Dakshinamurthy, Kishan Sullia

**Affiliations:** 1 Department of Bio-sciences, Mangalore University, Mangalagangotri, Mangaluru, India; 2 Santhathi Centre for Reproductive Medicine, Near Mangalore Nursing Home Upper Bendoor, Mangaluru, India

**Keywords:** Semen, pH, Count, Motility, Correlation, Semen Analysis

## Abstract

**Objective:**

The current study was aimed at correlating semen pH with motility and count to understand the significance of semen buffering system.

**Methods:**

The semen samples were collected from men who visited the clinic with infertility problems. Determination of semen pH, sperm motility and count were done according to the WHO laboratory manual, 2010 standards. The Mann-Whitney U-test was applied for statistical significance. The Pearson product moment correlation coefficient was used to measure the degree of linear relationship between the semen parameters.

**Results:**

For all patients (n=310) the mean±SD pH was found to be 8.4±0.3, with a range from 6.9 to 9.5. There was a significant and positive correlation between total motility and pH r =0.0591 (*p*<0.00001); volume and pH r=0.0582 (*p*<0.00001); Sluggish Progressive Motility (SPM) and pH, r = 0.0529 (*p*<0.00001); Abstinence and pH, r=0.0016 (*p*<0.00001). Negative correlation was noted between pH and total count r= -0.025 (*p*<0.00001); Rapid Progressive Motility (RPM) and pH r = -0.0776 (*p*<0.001), Non Motile (NM) spermatozoa and pH r=-0.00132 (*p*<0.00001).

**Conclusions:**

There were correlations between seminal pH and two important fertility parameters, viz., motility and count, indicating that the semen buffering system plays a vital role in maintaining the overall seminal quality, which is of clinical relevance.

## INTRODUCTION

We know that pH is a very important factor in maintaining the integrity of biomolecules and physiological functions. It is the same for semen, wherein pH plays an essential role in maintaining the functionality of spermatozoa during fertilization. As per the WHO guidelines (2010), 7.2 to 8.0 is the pH range required for a healthy semen. Based on statistics, it has been reported that nearly one in six couples are infertile, of which nearly half are associated with male factors (Brugh & Lipshultz, 2004). Earlier studies indicate that over 85% of men with infertility have the capacity to produce spermatozoa, but they are unable to fertilize an egg (Wolters-Everhardt *et al.,* 1986). Among various clinical manifestations of male infertility, decreased sperm motility (Asthenozoospermia) and reduction in sperm count (Oligozoospermia) are important ones to take into consideration (Wolters-Everhardt *et al.*, 1986; Haugen & Grotmol, 1998).

Since spermatozoa are the only human cells that perform a function outside the body from which they are produced, its environment plays a pivotal role during the transit and in the development of spermatozoa’s fertilizing ability (Hamamah *et al.*, 1996). The role of seminal plasma with its various constituents, particularly ions, in maintaining the buffering system with normal pH range, is important for other semen parameters, including motility and count. Seminal plasma is a mixture of secretions from testes, epididymis and accessory sex glands. Seminal plasma contains HCO^3-^/CO_2_, inorganic ions, organic acids, sugars, lipids, steroids, amino acids, polyamines, nitrogenous bases and proteins; which altogether contribute to the semen buffering system (Wolters-Everhardt *et al.,* 1986).

Semen pH has a very high buffering capacity, much higher than that of most other fluids in the body. The pH of human semen was a matter of debate. The pH of the seminal fluid may play a significant role in sperm function, the normal pH of seminal plasma is between 7.2 and 7.8 (WHO, 2010). An acidic ejaculate with pH less than 7.2 may be an indication of blockage of seminal vesicles, while that with an alkaline pH of about 8.0 is usually associated with infections (WHO, 2010). The time factor in determining the semen pH has been studied with a conclusion that there is a negative correlation between length of time and variation in pH owing to loss of CO2 after ejaculation (Makler et al., 1981; Wolters-Everhardt *et al.*, 1986). Earlier studies (Shedlovsky et al.,1942; Searcy & Simms, 1967] demonstrated that there is correlation between aging of whole semen fluid and significant reduction in pH, owing to fructolysis and the production of lactic acid.

Measurement of pH in the ejaculate is a part of the basic semen analysis. The pH of the ejaculate is primarily dependent on the basic seminal vesicle secretions and the more acidic prostatic secretions. More than 60% of the ejaculate volume originates from the seminal vesicles, whereas the prostate contributes with about 30% (WHO, 1992). The high content of inorganic phosphate and proteins in seminal plasma provide a considerable buffer capacity (Mann, 1964). Several inflammatory conditions, particularly of the prostate or seminal vesicles, as well as agenesis of vas deferens and seminal vesicles, may result in pH values outside the normal range. According to the latest version of the World Health Organization laboratory manual (WHO, 2010), the normal values for pH in liquefied semen are between 7.2 and 7.8, whereas in a previous version of WHO clinical manual (WHO, 1992) the normal range of values is from 7.2 to 8.0. Other laboratory handbooks in semen analysis state pH of normal semen to be in the range of 7.9-8.1 (Jequier & Crich, 1986) or 7.2-8.2 (Mortimer, 1994).

Cities located at sea level on the coast had higher pH levels (more than 8.0 / alkaline); whereas other laboratories in the non-coastal cities generally reports of pH values of 7.2 – 7.8. There are reports on the role of pH in determining the other seminal parameters. However, a literature survey reveals that reports on the correlation of pH with other parameters with special reference to the motility and count are very scanty (Haugen & Grotmol, 1998; Hamamah et al., 1996). With this current lacuna, and the hypothesis that semen pH plays an important role in determining fertility factors, including motility and count, the present study was taken up. The current study was aimed at correlating semen pH with motility and count, to understand the semen buffering system significance.

## MATERIALS AND METHODS

The Institutional Ethical Committee of Mangalore University (Cert. No. approved the study MU/AZ/187A/IHEC/2015-2016, dated on 22/06/2015). A total of 310 semen samples from the subfertile male patients were collected from June 2018 to December 2018 (Monsoon to Post Monsoon) from the patients who were native of Dakshina Kannada district who visited the Santhathi Centre for Reproductive Medicine, of the mean age of 34.5± 6.5. Prior to the study, a written consent was obtained from each participant.

Semen Samples were obtained by masturbation after at least 3 days of abstinence. Aspermic (those unable to produce the semen sample), subjects with erectile dysfunction and Obstructive Azoospermia and those who underwent antioxidant medication were excluded from the study. Only those samples produced for the first time from the subjects were considered for the study. The samples were ejaculated into sterile containers (Tarsons) and allowed to liquefy for at least 30 minutes before the analysis. Physical parameters such as volume, viscosity, color and microscopic parameters such as sperm count, motility (Grade A, B, C & D), were measured according to the World Health Organization’s (WHO, 2010) criteria. The Semen pH paper (MERCK 1730 PCLBL.0313) measured the pH.

### Statistical Analysis

Statistical analysis of the data was performed using the S-Plus 2000, MINITAB Statistical Software (Version 13.31; Minitab Inc.) The Mann-Whitney U-test (nonparametric test) with p<0.05 was considered statistically significant. The Pearson product moment correlation coefficient was used to measure the degree of linear relationship between the semen parameters.

## RESULTS

A total of 310 semen samples were assessed for the semen parameters such as total motility, total count, rapid progressive motility (RPM), sluggish progressive motility (SPM), non-motility (NM), abstinence (Abs) and pH. Our study included different conditions, like *Normozoospermia* (44%), with a high number followed by *Oligo-Astheno-Teratozoospermia* (OAT) (18%). The semen pH was compared to the semen parameters irrespective of the infertile condition. The mean± standard deviation (SD) age of patients in our population was 34.2±6.5 years. The mean±SD duration of abstinence before production of the specimen was 3.9±3.4 days and the mean±SD time from specimen production to analysis was 30.2±17 minutes. For all patients (n=310) the mean±SD pH was 8.4±0.3, with a median of 8.2. The range was 6.9 to 9.5, with pH.

**Figure 1:**
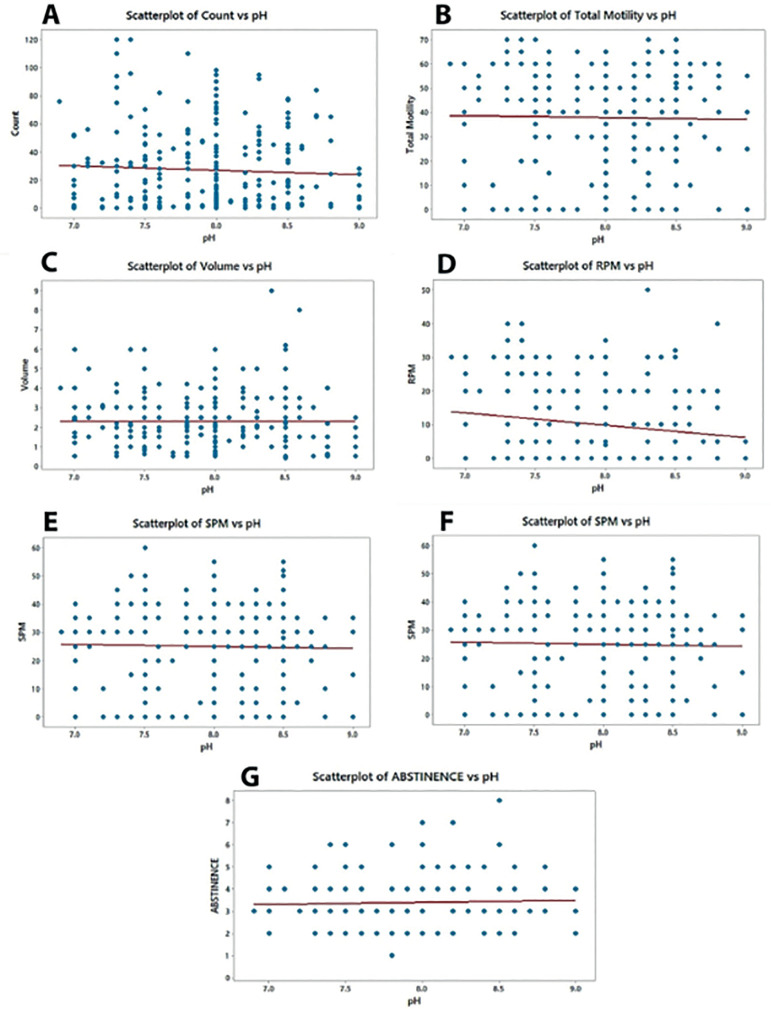
Scatter graphs illustrating the associations between Semen pH with other parameters. Individual data points are shown and linear regression lines indicate the relationship between the variables. There were significant and positive correlations between Total motility and pH (B: p<0.00001 r=0.0591), Volume and pH (C: p<0.00001, r=0.0582), SPM and pH (E: p<0.00001, r=0.0529), Abstinence and pH (G: p<0.00001, r=0.0582). There were significant and negative correlations between Total Count and pH (A: p<0.00001; r= -0.0250, RPM and pH (D: p<0.00001; r= -0.0776), NM and pH (F: p<0.00001, r= -0.00132)

A significant and positive correlation was seen between Total motility and pH r=0.0591; p<0.00001(Fig 1B), Volume and pH r= 0.0582, p<0.00001(Fig 1C), SPM and pH, r= 0.0529, p<0.00001(Fig 1E) Abstinence and pH, r=0.0016, p<0.00001(Fig 1G). However, relatively weak negative correlations between pH and Total Count and pH r= -0.025, p<0.00001(Fig 1A), RPM and pH r= -0.0776, p<0.0010 (Fig 1D), NM and pH r= - 0.00132, p<0.00001 (Fig 1F).

## DISCUSSION

The seminal plasma is derived primarily (50%-80%) from the seminal vesicles, with a smaller fraction (13%- 30%) contributed by the prostate. The Cowper and Littre glands provide an additional small percentage. The basic seminal vesicle secretions and the prostatic secretions, which may have a pH between 6.5 and 7.2, determine the pH of the ejaculate predominantly (WHO, 2010). With advancing age or infection this fluid may become more basic (Searcy & Simms, 1967) the normal pH of semen has been defined as ranging from 7.2 to 8.0. The mean (±SD) age of patients in our population was 34.5 ± 6.5 years. The mean (±SD) duration of abstinence before production of the specimen was 3.5 ±3.4 days and the mean (±SD) time from specimen production to analysis was 30.2 ± 15 minutes. All semen samples had an average pH of 8.2, with only 48% of the specimens falling within the supposedly normal range. Haugen & Grotmol (1998) also noted that the semen pH in their population was consistently higher than the WHO’s reference values, although they only studied 207 patients; both pH paper and a pH meter were used to analyze each sample. They found semen pH to be 8.2 with a pH meter and 8.4 with pH paper. Another group reported elevated semen pH among young healthy medical students (Haugen & Grotmol, 1998). Furthermore, there was no correlation between pH and the date of analysis through the 5-year study period, so it is unlikely that pH was affected by changes in lots of pH paper or changes in technicians during that period. Several inflammatory processes of the prostate and seminal vesicles are thought to alter semen pH, and according to the WHO infection should be suspected if the pH exceeds 7.8. (Hamamah et al.,1996; Mann, 1964) In light of our results, this recommendation could possibly lead to over diagnosis of infection and other inflammatory processes. It is interesting that the WHO manual states that the optimal pH for sperm migration and survival in the cervical mucus is 7.0 to 8.5. Although our findings confirmed those of Haugen & Grotmol (1998), we also extended their findings by demonstrating that the mean semen pH among patients with normal sperm parameters was not different from that among those with abnormal sperm parameters. Furthermore, a subgroup of patients with proved fertility in the same cycle in which the pH was measured also had the same high range of semen pH. It should be noted, however, that this subgroup could not be construed as a normal fertile population because the sperm was washed and inserted directly into the uterine cavity, as part of the intrauterine insemination procedure. This finding does demonstrate, however, that exposure to high pH semen does not preclude the functional potential of sperm.

Our study shows that the sample volume is positively correlated with the pH (r = 0.0582, *p*<0.00001), which clearly suggests that proper secretions from prostate as well as seminal vesicles in an appropriate volume balances the pH of the sample, which is very vital for the normal functioning of the human spermatozoa. Total motility (r = 0.0591; *p*<0.00001) showed a positive correlation with pH, however the total count (r = -0.025, *p*<0.00001) showed a negative correlation with the pH. Likewise Sluggish progressive motile (SPM) (r=0.0529, *p*<0.00001) showed a positive correlation; whereas, Rapid progressive motile (RPM) (r = -0.0776, *p*<0.0010) showed a negative correlation with the pH.

At present, we cannot explain the discrepancy in the published pH values, since the measurements in the various reported experiments (Homonnai et al., 1978; Bhushan et al., 1978; Chaudhari et al., 1990; Cooper et al., 1991; Blackwell & Zaneveld,1992; Sofkitis & Miyagawa 1993; Calderon et al., 1994) seem to have been performed under the conditions recommended by the WHO (2010). The results of the present study are based on a limited number of subjects (n=310), which may also include fertile men. The pH range derived from the present study deviates from the pH range defined by WHO; therefore, further studies are required to provide an insight into the role of pH in maintaining the quality of semen for fertility taking geographical regions into consideration.
